# Supplementary material for: Transcriptional Trajectories in Mouse Limb Buds Reveal the Transition from Anterior-Posterior to Proximal-Distal Patterning at Early Limb Bud Stage
Source: J Dev Biol. 2020 Dec 7;8(4):31. doi: 10.3390/jdb8040031 (PMC7768367; doi:10.3390/jdb8040031)
Supplement: Supplementary file 1 [file jdb-08-00031-s001.pdf]

Journal of Developmental Biology

MDPI

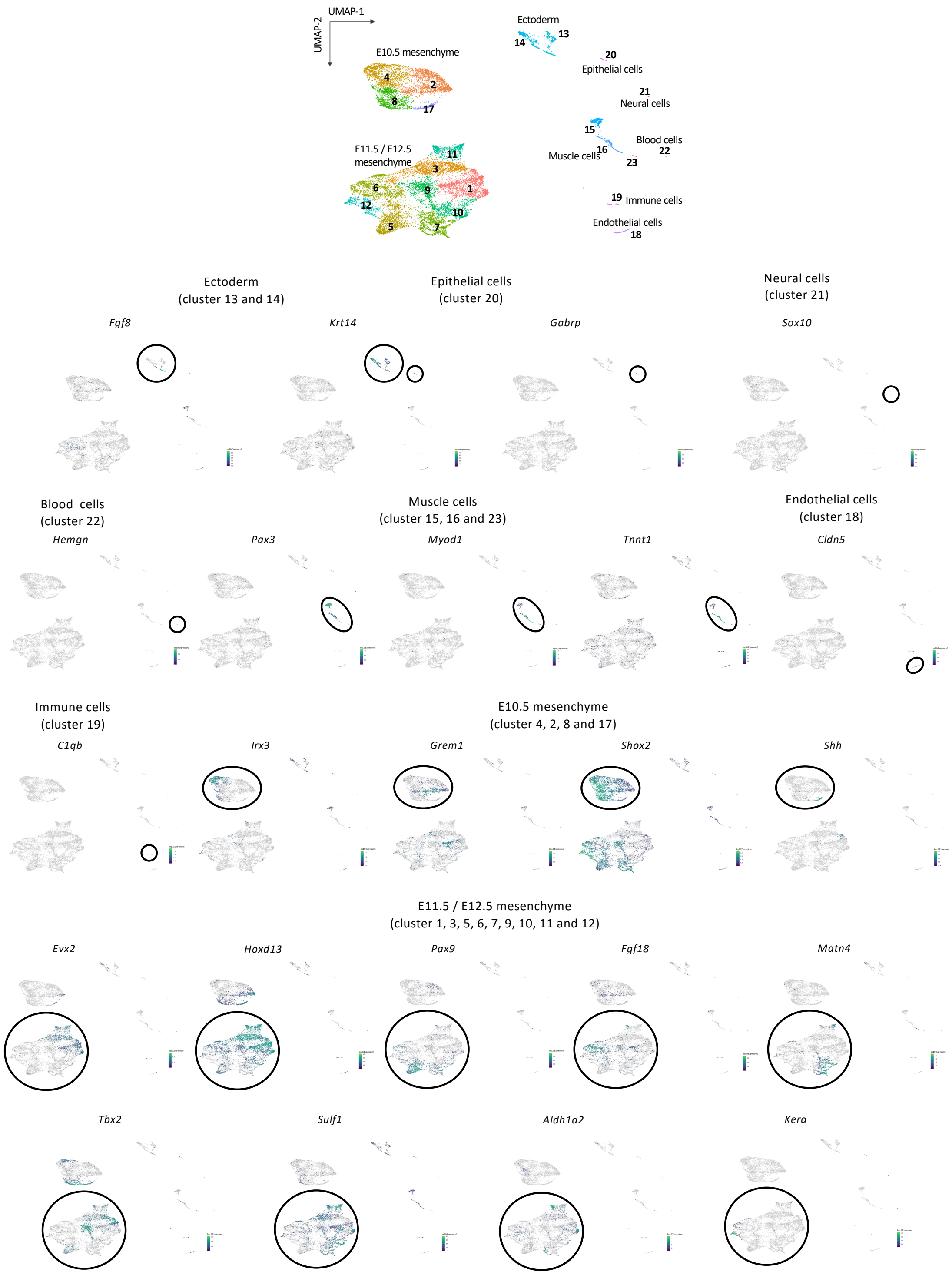

Figure S1. Expression profiles of the different gene markers in the developing mouse limb bud. UMAP visualization of 18,935 mouse forelimb buds cells at E10.5, E11.5 and E12.5 colored by cluster identity from Leiden clustering. The clusters were annotated based on the identification of marker genes. The UMAP visualization of limb bud cells at E10.5, E11.5 and E12.5 expressing marker gene for each cluster is shown. Color scale indicated the scaled expression of each gene.

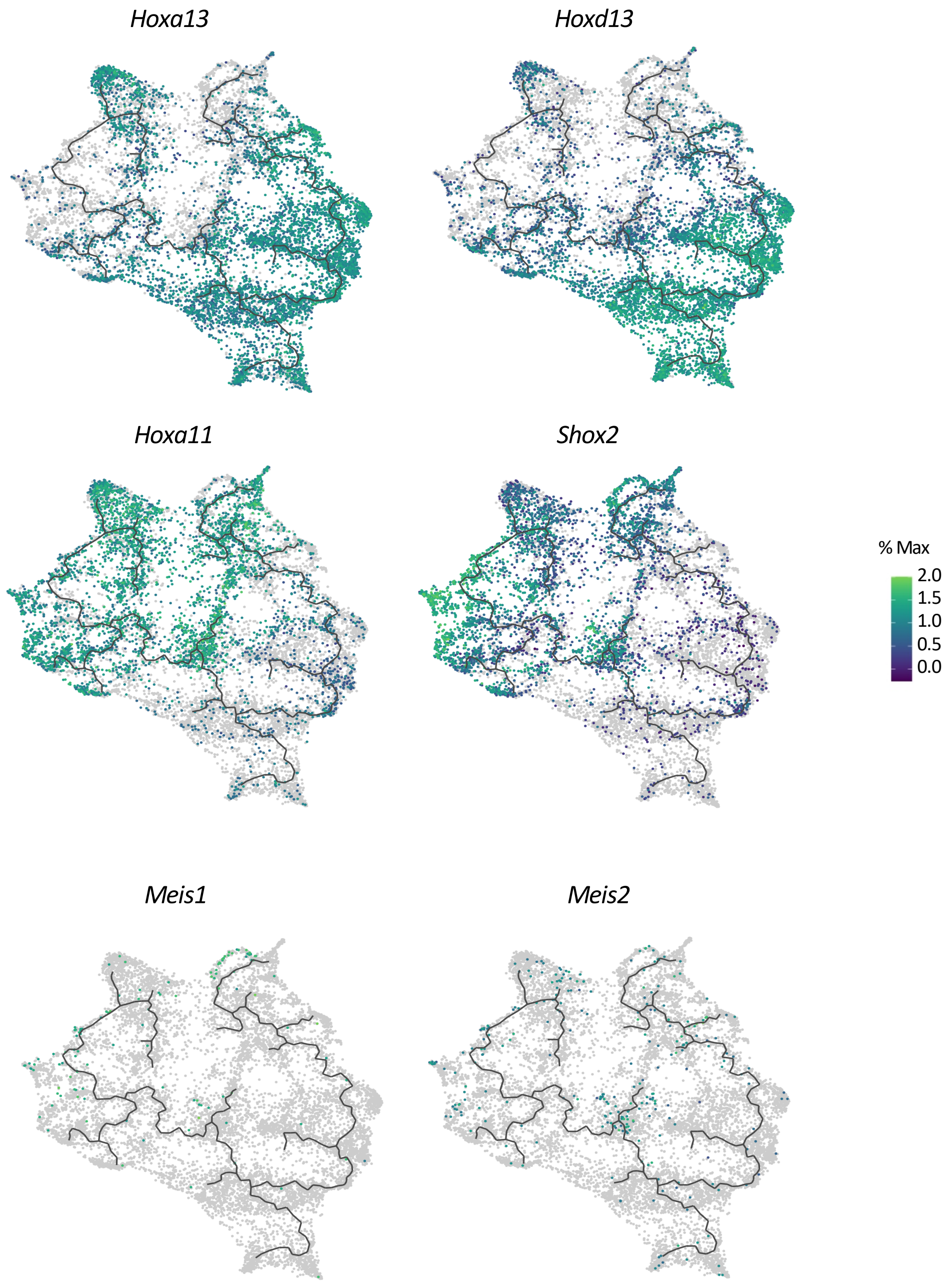

Figure S2. Prevalence of the distal limb tissue as compared to proximal tissue. UMAP visualization of the mesenchymal cells expressing distal genes *Hoxa13* and *Hoxd13* and proximal genes *Hoxa11*, *Shox2*, *Meis1* and *Meis2* at E11.5 and E12.5. Color scale indicated the scaled expression to percent of the maximum expression of all genes at E11.5 and E12.5.

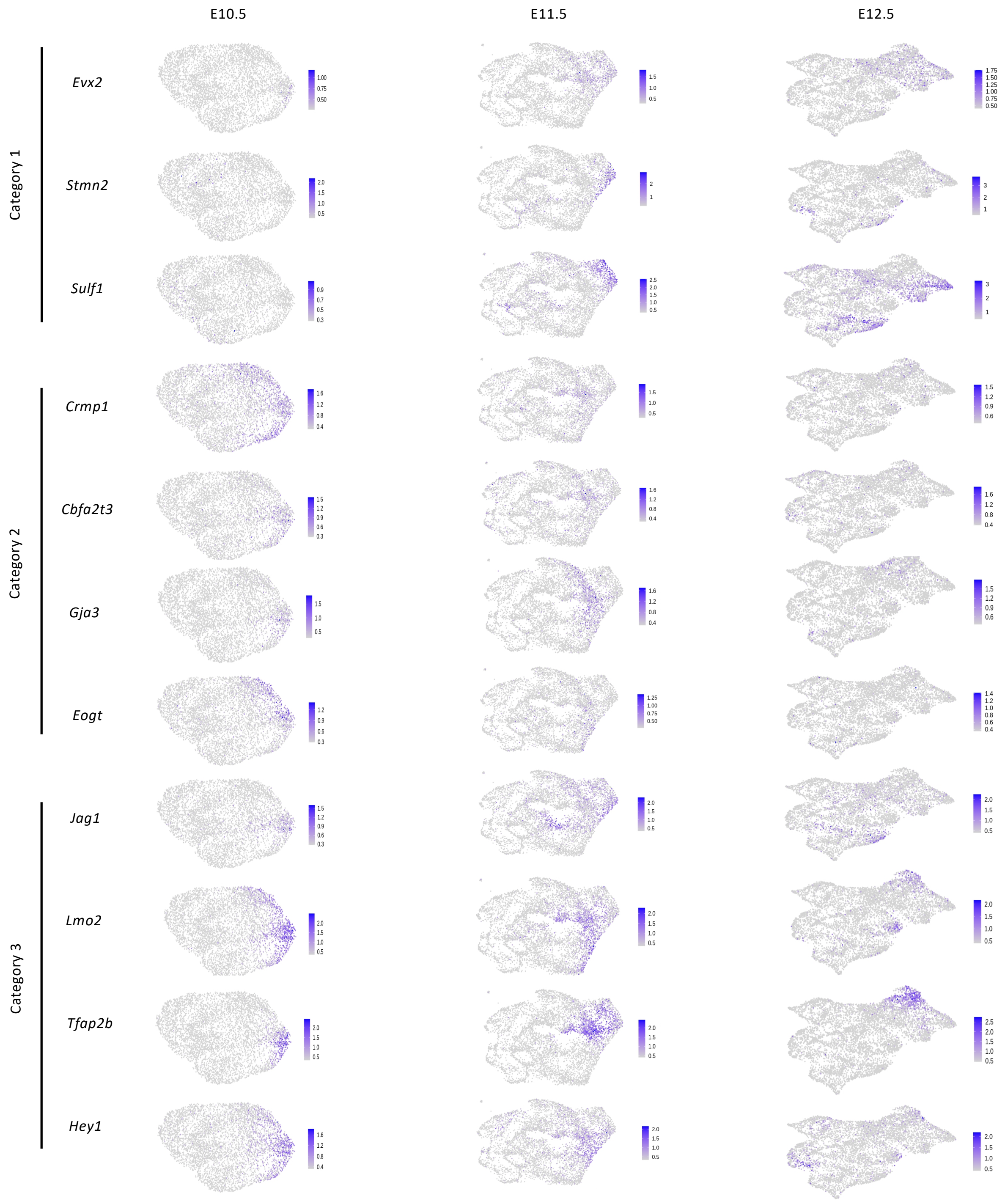

Figure S3. Examples of dynamic expression of distal genes during limb bud development. UMAP visualization of the mesenchymal cells expressing the distal genes *Evx2, Stmn2* and *Sulf1* from category 1; *Crmp1, Cbfa2t3, Gja3* and *Eogt* in category 2; *Jagged1, Lmo2, Tfap2b* and *Hey1* belonging to category 3 in E10.5, E11.5 and E12.5 WT mouse forelimb buds. Color scale represents the scaled expression for each gene.

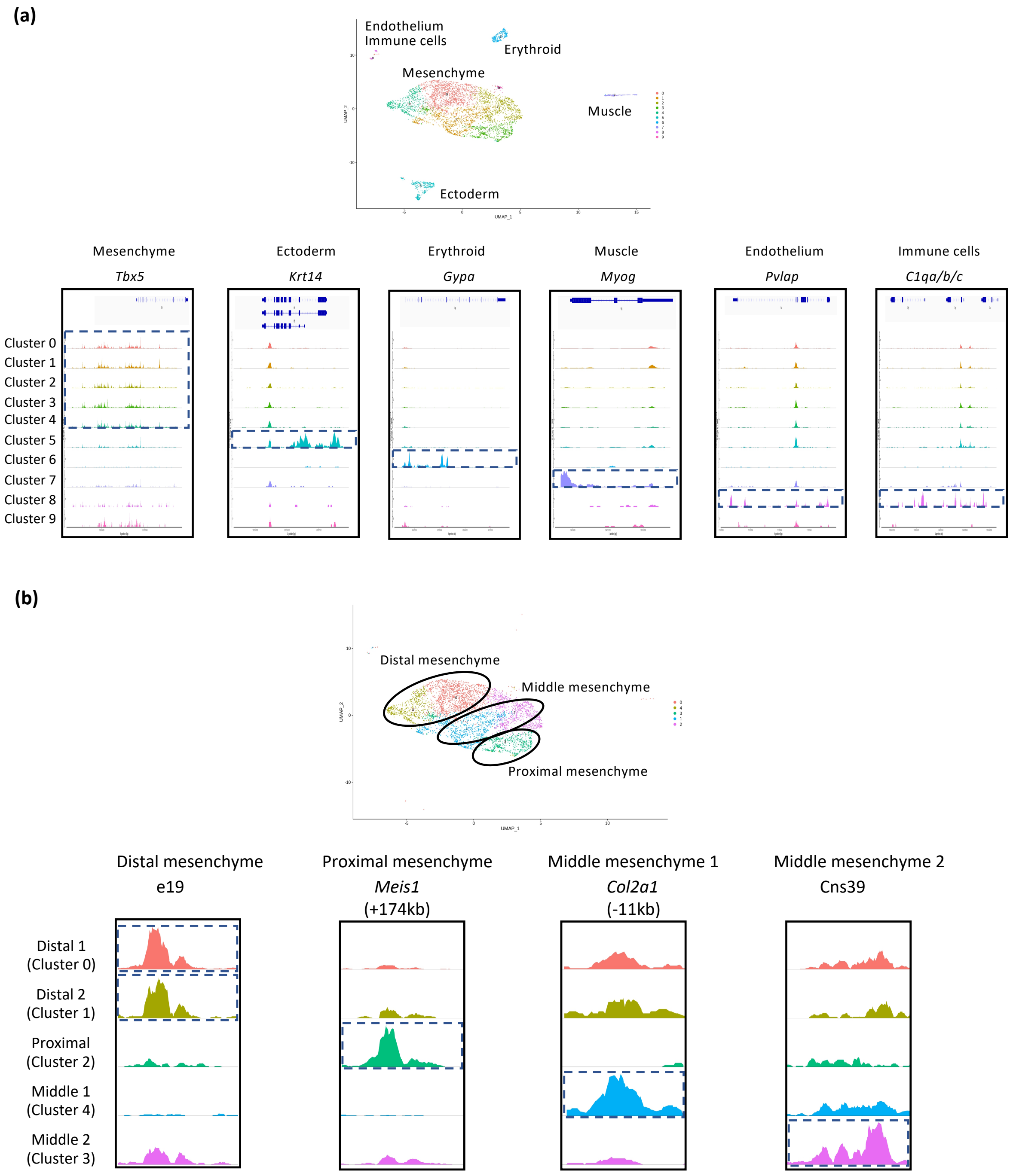

Figure S4. Annotation of the clusters identified by scATAC-seq. (**a**) (**b**) Genome views of scATAC-seq signals (Signac) at one selected marker per cluster for the non mesenchymal clusters (**a**) and for the mesenchymal clusters (**b**) in E11.5 mouse forelimb buds.

(a)

	E10.5<br>5,168 cells	E11.5<br>5,524 cells	E12.5<br>6,430 cells
Hoxa13-;Gfp+	15 cells	257 cells	1,080 cells
Hoxa13-;Hoxd13+;Gfp+	5 cells	149 cells	771 cells
Hox13-;Gfp+	10 cells	108 cells	309 cells

(b)

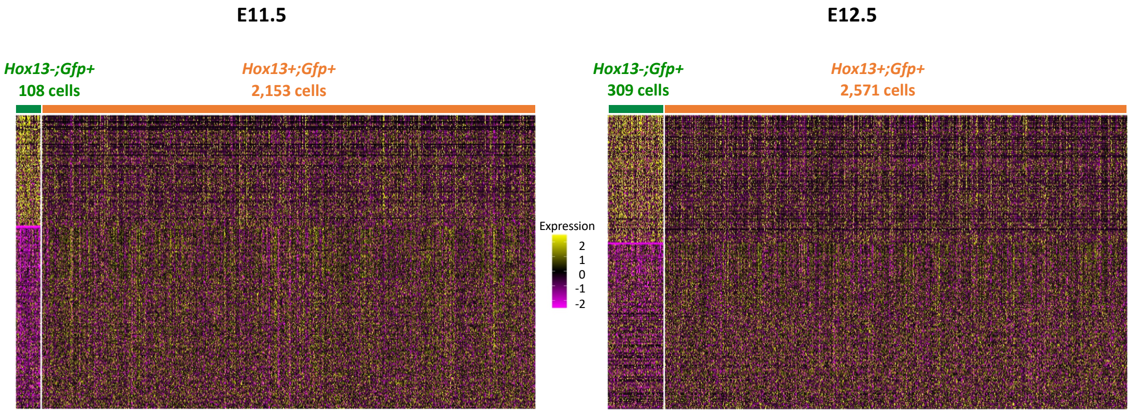

(c)

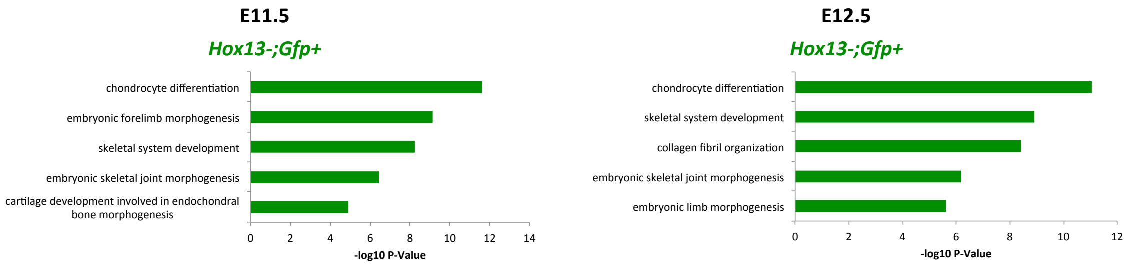

Figure S5. Analysis of the *Hox13-;Gfp+* cell population in the developing limb mesenchyme. (**a**) Table showing the number of cells for each genotype. (**b**) Heatmap showing the significant (*p*-Value < 0.05) gene markers for *Hox13-;Gfp+* and *Hox13+;Gfp+* cell populations. (**c**) DAVID analysis showing the enriched GO terms for biological processes for gene markers of the *Hox13-;Gfp+* cell population.
